# Physician modification of a Gore endograft for accessory renal artery preservation

**DOI:** 10.1016/j.jvscit.2026.102168

**Published:** 2026-02-05

**Authors:** Niraj Balakrishnan, Indrani Sen

**Affiliations:** Department of Vascular and Endovascular Surgery, Mayo Clinic Health System, Eau Claire, WI

**Keywords:** Accessory renal artery preservation, Fenestrated endovascular repair, Infrarenal abdominal aortic aneurysm, Off-label device modification, Physician modified endograft, Renal function preservation

## Abstract

Accessory renal arteries arising within the proximal seal zone complicate endovascular repair of infrarenal abdominal aortic aneurysms, particularly in patients with chronic kidney disease. We report the case of 76-year-old man with diabetes, hypertension, coronary artery disease status post coronary artery bypass graft, and stage 3a chronic renal failure (estimated glomerular filtration rate, 49-55 mL/min/1.73 m^2^) who presented with a 5.5-cm infrarenal abdominal aortic aneurysm with gradual growth on surveillance. The infrarenal neck measured 22 to 24 mm in diameter and 27 mm in length, with circumferential wall calcification and atheroma. Two accessory renal arteries originated in the middle of the seal zone, each supplying a significant proportion of the renal parenchyma. The main renal arteries were small (4.1/4.2 mm at the origin) and somewhat diseased with ostial calcification. Open repair with possible suprarenal clamp was considered higher risk for renal dysfunction. Endovascular aneurysm repair using a physician-modified endograft with fenestrations created using a punch biopsy device was performed. The procedure was technically successful, with no type I or III endoleak and no change in the estimated glomerular filtration rate at the 2-month follow-up.

This is a case of 76-year-old man with diabetes, hypertension, coronary artery disease status post coronary artery bypass grafting and stage 3a chronic renal failure (estimated glomerular filtration rate, 49-55 mL/min/1.73 m^2^) who presented with a 5.5-cm infrarenal abdominal aortic aneurysm (screen detected in 2018, with gradual growth to present size). Centerline measurements (Terarecon) (Supplementary Fig 1, online only) revealed the infrarenal neck measured 22 to 24 mm in diameter and was in total 27 mm in length, with no significant angulation circumferential wall calcification and atheroma. The aortic bifurcation measured 20 mm in diameter. The right common iliac measured 15 mm and the left common iliac12 mm in diameter; both external iliac arteries measure 7 to 8 mm in diameter.

There were two accessory renal arteries originating in the middle of this seal zone, one on either side, each supplied a significant proportion of the renal parenchyma. The main renal arteries (Supplementary Video, online only) were small (4.1/4.2 mm at the origin) and somewhat diseased with ostial calcification. The accessory renal arteries measured 3.1 and 2.8 mm at the ostia. The infrarenal neck below these accessory renals was conical (22-27 mm) (Supplementary Fig 1, online only).

Although accessory renal sacrifice is was traditionally considered reasonable when <3 mm,^1^ intentional accessory renal artery (ARA) coverage during endovascular repair is associated with a threefold increase in acute kidney injury and renal function deterioration.^2^ Open repair was considered; however, owing to circumferential wall calcification and possible requirement of suprarenal clamp, there would have been a higher risk of renal dysfunction.^3^ The patient declined open repair and provided informed consent for endovascular aneurysm repair (EVAR) using a physician-modified endograft use (off label) in a shared decision-making model, and for this publication.

Because the main renal arteries were small and diseased and the ARAs supplied a significant proportion of the renal parenchyma, ARA preservation was necessary. Graft considerations included consideration of existing EVAR and fenestrated EVAR devices. A suprarenal seal would necessitate incorporation of the diseased small main renal and accessory renal arteries with scallops/fenestrations and require stents into both small diseased main renal arteries. This procedure would have had a little higher risk of renal dysfunction from multiple possible factors: atheroemboli from the mild atheroma at the aortic neck, small size of the renal and accessory renals, and need for possible use of coronary covered stents. Based on the distance of the accessory and main renal arteries from the proximal landing zone, a patient-specific design would not be feasible. When considering suprarenal vs infrarenal fixation, if there were no ARAs/renal dysfunction, the infrarenal neck would be adequately sealed with a traditional infrarenal sealing device. Thus, in this situation, physician modification was considered the best treatment option.

Based on centerline measurements a Gore physician-modified endograft was feasible. Consideration of modification of an alternate device was considered, but owing to the need for two fairly small accessory renal fenestrations (no difficulty planning in relation to stitch line), avoiding the proximal fixation barbs, and avoiding need for another stent component, this graft was preferred for ease of modification. Overall, reports of graft modification using this device are less frequent, because planning modifications around the stitch line can be restrictive for positioning of multiple fenestrations.^4^, ^5^, ^6^, ^7^ The Supplementary Video (online only) shows the steps involved in back table modification using a punch biopsy device instead of heat^8^/ophthalmic cautery to create the fenestrations.

The inner stylet is retained to maintain device integrity. The shipping sheath cover is retracted over the body of the device. The second stent is secured with a silk tie for controlled deployment. The white outer handle is partially deployed until the gray knob is visible. Next, the gray knob is rotated clockwise to fully constrain the proximal end of the endograft. During this step, the assistant's hand stabilizes the first stent to prevent peeling back the secondary sleeve. With the stitch line visible, the white handle at the distal end is pulled slowly to allow each suture to be pulled back to just beyond the point where the stent has been tied down with the silk. This white suture is somewhat difficult to see; this step needs to be done very slowly. Next, a 4-0 Prolene needle is used to tease up the white suture loop. The loop is threaded through the eye of the needle, taking care not to pierce the fibers, because this may render the device unusable. Once the loop is threaded, the opposite end of the needle is secured with a clamp. Using a nerve hook, the outer sleeve deployment fiber on the opposite side of the loop is located and this fiber is pulled up to retrieve the slack from the handle. With gentle backward traction on the nerve hook, the deployment fiber is locked with seven to eight Aberdeen knots. Now, at the back handle, the white part is withdrawn until the larger loop tightens down onto the nerve hook. The nerve hook is then withdrawn to engage the tightening ridge with its proximal tip. It is kept in place until the final loop tightens, and only then is the nerve hook taken out.

Now, the silk tie around the second stent row is removed, the anchoring string is cut and peeled back like a banana peel, and this primary sleeve is cutoff. The top of the secondary sleeve is anchored with a 5-0 Prolene stitch to the graft and set aside with a rubber shoe. This Prolene stitch is removed later. The gray handle is rotated counterclockwise to open the proximal graft. As this is being done, the sleeve is pinched forward to avoid bunching of the secondary sleeve below the stents that are opening up; after this, the graft material is opened with the thumb. Fenestrations are planned to avoid damaging the stitch line; this step is critical. We used a biopsy punch to create a fenestration supporting the material from the underside with a piece of gauze and gentle outward pressure. In our case, this technique was extremely successful, and the fenestration can be dilated up to a final size of 6 to 8 mm using a hemostat. Absence of heat sealing on the edge is not a problem, because the graft edge does not fray. The fenestration is reinforced with a marker wire in standard fashion. Next a 2-0 Prolene suture is taken through the graft material and a stent in the distal end of the device. These Prolene sutures are externalized through the shipping sheath to apply backward traction on the device during resheathing. Resheathing is a two-person task. The assistant holds backward traction using the Prolene suture, as the surgeon pinches down the stents and slides the shipping sheath over. The first stent is usually well-constrained, so it slides in easily; the second stent row may need to be pinched down with two hands from the top as the shipping sheath is slid on to ensure that the stents are not damaged. At the proximal end, resheathing is achieved by sliding the shipping sheath over the anchors. These anchors are made of nitinol and will temporarily fold upward and spring back into place when unsheathed. The end of the shipping sheath is cut down with an 11 blade, and then it is pulled back down over the nose cone. The device is then loaded on with the shipping sheath, and the femoral DrySeal (W. L. Gore & Associates) flushed thoroughly so that the window is clear. During introduction the shipping sheath is advanced into the DrySeal (W. L. Gore & Associates) until the sheath can be seen in the clear window. The device is then introduced into the patient and the repair completed in the standard fashion. In this patient, alignment of fenestration was performed with a limited diagnostic angiogram; the ARAs were not stented. The procedure was technically successful; there were no complications during deployment, with no type I or III endoleaks at completion, and no change in the estimated glomerular filtration rate at the 2-month follow-up (Supplementary Fig 2, online only). Reports of non-investigational device exemption use are necessary to further improve the safety of future patients.^7^

## Funding

None.

## Disclosures

None.

## Figures and Tables

**Supplementary Figure 1:**
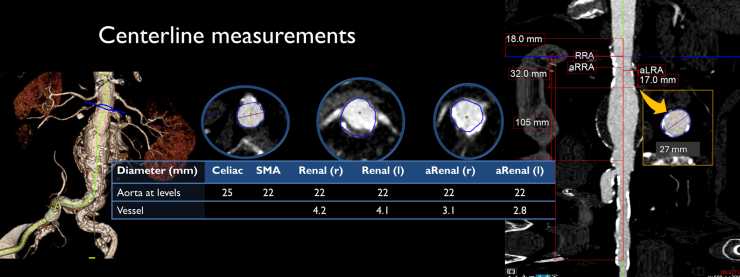
Centerline measurements (Terarecon) revealed the infrarenal neck measuring 22 to 24 mm in diameter and was in total 27 mm in length, with no significant angulation circumferential wall calcification and atheroma. There were two accessory renal arteries originating in the middle of this seal zone, one on either side, each supplied a significant proportion of the renal parenchyma. The main renal arteries (Supplementary Video, online only) were small (4.1/4.2 mm at the origin) and somewhat diseased with ostial calcification. The accessory renal arteries measured 3.1 and 2.8 mm at the ostia. The infrarenal neck below these accessory renals was conical (22-27 mm).

**Supplementary Figure 2:**
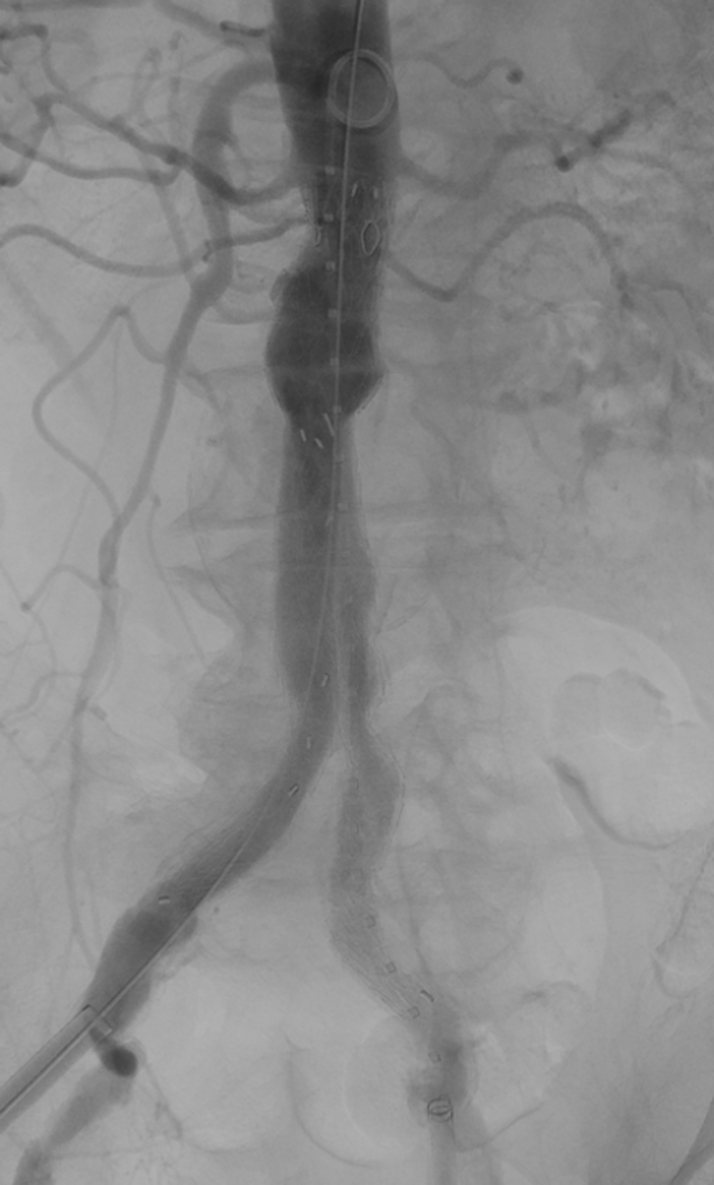
The procedure was technically successful, there were no complications during deployment, with no type I or III endoleaks at completion, and no change in GFR at the 2-month follow-up.
